# The acute effects of whole-body vibration on motor unit recruitment and discharge properties

**DOI:** 10.3389/fphys.2023.1124242

**Published:** 2023-02-21

**Authors:** E. Lecce, S. Nuccio, A. Del Vecchio, A. Conti, A. Nicolò, M. Sacchetti, F. Felici, I. Bazzucchi

**Affiliations:** ^1^ Laboratory of Exercise Physiology, Department of Movement, Human, and Health Sciences, University of Rome “Foro Italico”, Rome, Italy; ^2^ Department Artificial Intelligence in Biomedical Engineering, Faculty of Engineering, Zentralinstitut für Medizintechnik (ZIMT), Friedrich-Alexander University Erlangen-Nürnberg, Erlangen, Germany

**Keywords:** WBV, HDsEMG, neuromuscular control, muscle strength, MU recruitment

## Abstract

**Introduction:** several studies have reported improved neuromuscular parameters in response to whole-body vibration (WBV). This is likely achieved by modulation of the central nervous system (CNS). Reduced recruitment threshold (RT), which is the % of Maximal Voluntary Force (%MVF) at which a given Motor Unit (MU) is recruited, may be responsible for the force/power improvements observed in several studies.

**Methods:** 14 men (25 ± 2.3 years; BMI = 23.3 ± 1.5 kg m^2^ MVF: 319.82 ± 45.74 N) performed trapezoidal isometric contractions of the tibialis anterior (TA) at 35-50-70 %MVF before and after three conditions: WBV, STAND (standing posture), and CNT (no intervention). The vibration was applied through a platform for targeting the TA. High-density surface electromyography (HDsEMG) recordings and analysis were used to detect changes in the RT and Discharge Rate (DR) of the MUs.

**Results:** Mean motor unit recruitment threshold (MURT) reached 32.04 ± 3.28 %MVF before and 31.2 ± 3.72 %MVF after WBV, with no significant differences between conditions (*p* > 0.05). Additionally, no significant changes were found in the mean motor unit discharge rate (before WBV: 21.11 ± 2.94 pps; after WBV: 21.19 ± 2.17 pps).

**Discussion:** The present study showed no significant changes in motor unit properties at the base of neuromuscular changes documented in previous studies. Further investigations are needed to understand motor unit responses to different vibration protocols and the chronic effect of vibration exposure on motor control strategies.

## 1 Introduction

In the last decade, increasing emphasis has been pointed to neuromuscular training to improve motor control in human movement. Among several strategies, the use of high-frequency mechanical oscillations to enhance neuromuscular properties, even called whole-body vibration (WBV), has been brought to the forefront. Numerous studies have additionally demonstrated improved performance in response to acute WBV, including increases in strength and power (Delecluse et al., 2003; Rees et al., 2009), motor coordination (Ness and Field-Fote, 2009), and postural control in different populations (patients, athletes, elderly etc.) (Orr, 2015). On the other hand, Kurt and Pekünlü (2015) have described a non-significant variation in squat jump, trunk flexion, and isometric leg strength performances after a WBV protocol. Moreover, Brent Feland et al. (2021) have reported no significant effects on vertical jump performance and electromechanical delay parameters. However, the reported improvements were speculated to be associated with enhanced neural excitation, likely achieved at the spinal or supraspinal level by central nervous system modulation (Cochrane et al., 2010). It has been suggested that any change may be caused by acute neural responses, such as altered motoneuron excitability, synergist and antagonist coactivation, spindle sensitivity, modifications in motor unit (MU) recruitment threshold, and MU synchronization (Cardinale and Bosco, 2003). However, potential neural mechanisms have not been utterly investigated.

The hypothetical reduction in the recruitment threshold of fast-twitch fibers may be responsible for the improvements observed and could explain the greater effect on mechanical power output by increased contractile speed (Pollock et al., 2012). Nevertheless, very few studies have investigated WBV effects on the MU recruitment threshold and the firing properties of single MUs. Among these, Pollock and colleagues have described an increase in the low-threshold MUs RT after acute WBV, although this did not appear to be related to changes in presynaptic inhibition. They have also found an opposite effect on higher threshold MUs, which they explained with the primary involvement of polysynaptic pathways, not much involved in low-threshold motor unit pathways. As the authors suggested, a limitation of their study was the small number of analyzed MUs (Pollock et al., 2012). It would have been valuable to study a greater number of MUs, particularly the high-threshold ones, but due to difficulties in identifying single MUs at higher forces, this was not possible. Nowadays, technological advances (i.e., high-density surface electromyography decomposition technique) have brought the possibility to identify the concurrent activity of several tens of MUs, in contrast to much smaller samples available in classic studies with indwelling recordings (Farina et al., 2016). Moreover, classic MU recording and analysis methods do not allow the same motor unit tracking across different experimental sessions. Therefore, there is limited experimental evidence on the adjustments in MU properties following WBV training. For the mentioned reasons, this study has been designed to investigate the acute effects of acute WBV exposure on MUs behavior. We hypothesize that cortical and subcortical facilitation can be achieved, accompanied by reduced spinal excitability after an acute bout of WBV, which points towards greater control of voluntary movement.

## 2 Materials and methods

### 2.1 Participants and ethical approval

Fourteen recreationally active male volunteers (mean ± SD; age, 25 ± 2.3 years; BMI = 23.3 ± 1.5 kg m^-2^; MVF: 319.82 ± 45.74 N) were enrolled in the study. The exclusion criteria included: a) history or signs of neurological or neuromuscular diseases; b) undertaken limb surgery. Participants signed an informed consent outlining experimental procedures and potential side effects before their involvement in the study. The experiments were conducted in accordance with the Declaration of Helsinki. The Ethics Committee of the University of Milan “Politecnico” approved the protocol (process number 09/2021).

### 2.2 Experimental design

**FIGURE 1:**
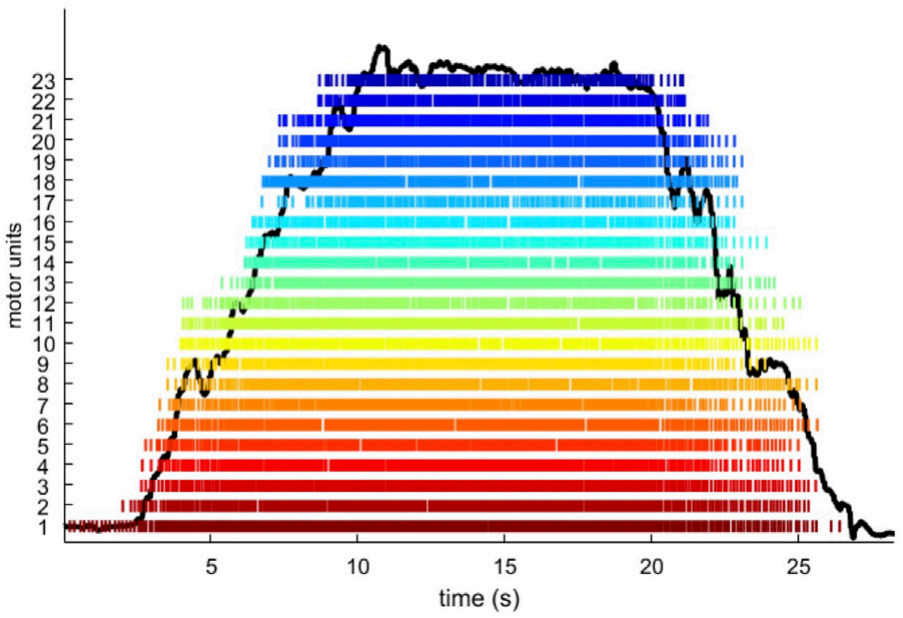
An example of a trapezoidal ramp. Representative trapezoidal ramp at 35% MVF with a rate of force increase of 5%MVF·s^1^ of participant number 11. Each stripe corresponds to a single motor unit, and each stick within the stripe corresponds to a motor unit firing.

Participants visited the laboratory on 4 days separated by at least 48 h. Within the first visit, participants familiarized with the experimental protocol, whereas during the remaining ones performed the three experimental sessions: a) whole-body vibration applied in a standing posture (WBV); b) maintaining the same standing posture as the WBV in the absence of the vibrating stimulus (STAND); c) no intervention (CNT). The experimental sessions were randomized across participants. The tibialis anterior (TA) of the dominant leg was chosen for analyses. The dominant leg was identified by asking participants which leg they use to kick a ball, as previously reported (van Melick et al., 2017; Nuccio et al., 2021b). TA muscle was chosen in accordance with previous studies using HDsEMG and experimental setup (del Vecchio et al., 2019). Before testing procedures, a standardized warm-up was done (3 × 30 low-intensity isometric dorsiflexions, 30 s rest), then, three maximal voluntary contractions (MVCs) separated by 180 s were performed to set the maximal voluntary force (MVF), which is the peak force value across the three contractions. MVCs were performed before the testing phase in each experimental session to set the MVF, according to the daily force output variability (Raphael et al., 2019). Once the MVF was set, participants performed isometric trapezoidal contraction trials (Figure 1) at three target forces (35-50-70%MVF) in a randomized order.

**FIGURE 2:**
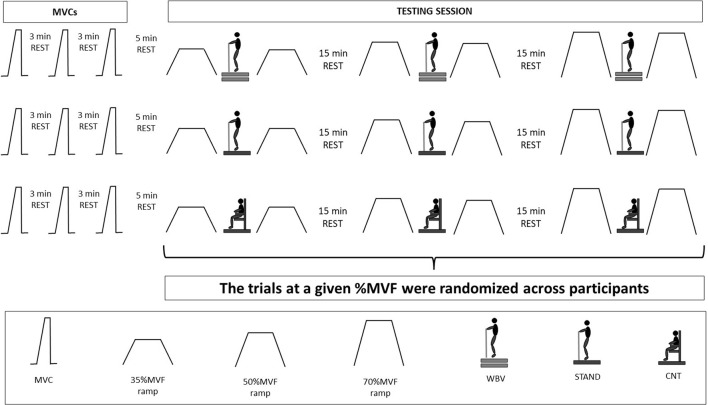
Experimental setup. Schematization of the experimental protocol. During each experimental session, participants performed three MVCs and three pre-post-trapezoidal contractions at each force target (35-50-70%MVF).

They were asked to exert force accurately matching the trapezoidal path displayed on a monitor placed 1 m from their eyes. Two trapezoidal contractions were performed at each target force (35-50-70%MVF) before and after each experimental condition, based on session day (Figure 2). Trials were separated one from another by 15 min rest to guarantee a complete acute vibrating effect disposal (Laudani et al., 2018). The participants were asked to refrain from strenuous exercise and caffeine consumption in the 48 h before the testing sessions.

### 2.3 Vibration, standing, and control conditions

During the WBV session, volunteers stood barefoot on a vibration platform in an upright position. The vibration frequency was set to 30 Hz and the amplitude to 4 mm (Cormie et al., 2006). The static body position was maintained in a forefoot stance with a knee angle of 10° (taking 0° as the anatomic position) and a plantarflexion angle of 120°. Participants were instructed to place their hands on the platform handle, directing their head and eyes forward, and distributing their weight equally on both feet. Once participants were correctly positioned, the vibrating stimulus started. A total of three vibrating stimuli of 1 min each, were delivered in a single WBV session, one per each target force (35-50-70%MVF). In the STAND session, participants were placed on the platform in the absence of the vibrating stimulus, maintaining the same position for 1 min. The STAND condition was provided to compare potential differences derived by position maintenance. During the control (CNT) session, participants remained seated for 2 min between trial contractions (Figure 2), which is the time needed to find the correct posture and prepare the experimental setup (roughly 1 min) plus 1 min provided in both WBV and STAND conditions.

### 2.4 Force signal recording

The experimental setup consisted of a vertically positioned custom-made ankle ergometer (OT Bioelettronica, Turin, Italy). Participants were seated on a chair with the dominant leg held in the ankle ergometer with straps at the foot, ankle, and knee. The hip and the knee were flexed at ∼90° and the ankle was placed at ∼100° of plantar flexion. The foot and the ankle were maintained with straps on an adjustable footplate connected in series with a calibrated load cell (CCt transducer s. a.s. Turin, Italy). The signal recorded with the force transducer was amplified (x200) and sampled at 2048 Hz with an external analog-to-digital (A/D) converter (OT-Bioelettronica, Turin, Italy). The force signal, recorded with OTbiolab software (OT-Bioelettronica), was synchronized with the electromyogram. Visual feedback of both the guided pattern and the performed force signal was displayed on a monitor positioned 1 m from the participants’ eyes.

### 2.5 Trapezoidal ramps and high-density surface EMG recordings

**FIGURE 3:**
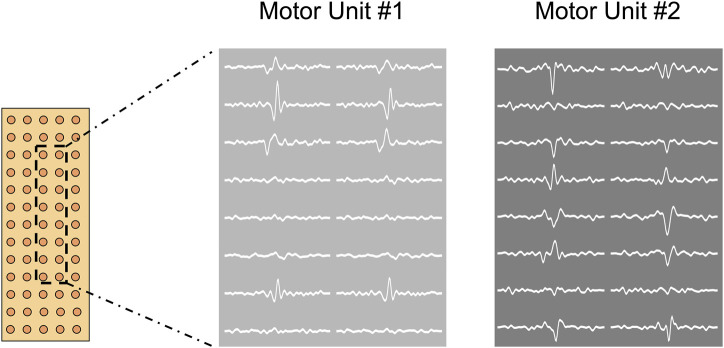
Graphical representation of a high-density EMG grid and signal. In this figure it is depicted an example of two different motor unit action potentials obtained from the decomposition process. Grids are made up of 64 electrodes (13 rows x 5 columns).

Participants performed trapezoidal ramps at three force levels (35-50-70%MVF) during isometric ankle dorsiflexions. Trapezoidal ramps consisted of a linear force increase to the target value at a rate of 5% MVF·s^-1^, 10 s of steady force at the target %MVF, and a linear force decrease back to the resting value at the same rate as the increasing phase. As a result, ramps had three different durations: 35%MVF of 24 s, 50%MVF of 30 s, 70%MVF of 38 s. HDsEMG signals were recorded from the Tibialis Anterior (TA) with two adhesive grids of 64 electrodes each [13 rows x five columns; gold-coated; diameter 1 mm; inter-electrode distance (IED) 8 mm; OT-Bioelettronica] (Figure 3).

After skin preparation (shaving, light skin abrasion, and 70% ethanol cleansing), the muscle perimeter was identified through palpation and marked by a surgical pen. The grids orientation was based on recordings from a 16-electrodes array (IED 5 mm, OT-Bioelettronica), identifying the TA innervation zone and estimating the direction of the fibers, as described in (del Vecchio et al., 2019). Once the innervation zone was found, one grid was placed over the TA distal portion, and the other was proximally placed, both parallelly to the lateral tibial margin using a disposable biadhesive with layer holes adapted to the HDsEMG grids (SpesMedica, Battipaglia, Italy). The innervation zone was located by identifying the point of inversion in the propagation direction of action potentials proximally (toward TA proximal tendon) and distally (toward TA distal tendon) along the electrode column. The foam layers holes were filled with a conductive paste (SpesMedica) to ensure skin-electrode contact. Reference electrodes were positioned on the styloid process of the ulna, on the tibial tuberosity, and medial malleolus. HDsEMG signals were recorded in monopolar mode and converted to digital data by a 16-bit multichannel amplifier (EMG-Quattrocento, 400 channel EMG amplifier; OT Bioelettronica; 3 dB, bandwidth 10-500 Hz). The HDsEMG signals were amplified (x150), sampled at 2048 Hz, and band-pass filtered (10-500 Hz) before being stored for offline analysis.

### 2.6 Force and HDsEMG analysis

**FIGURE 4:**
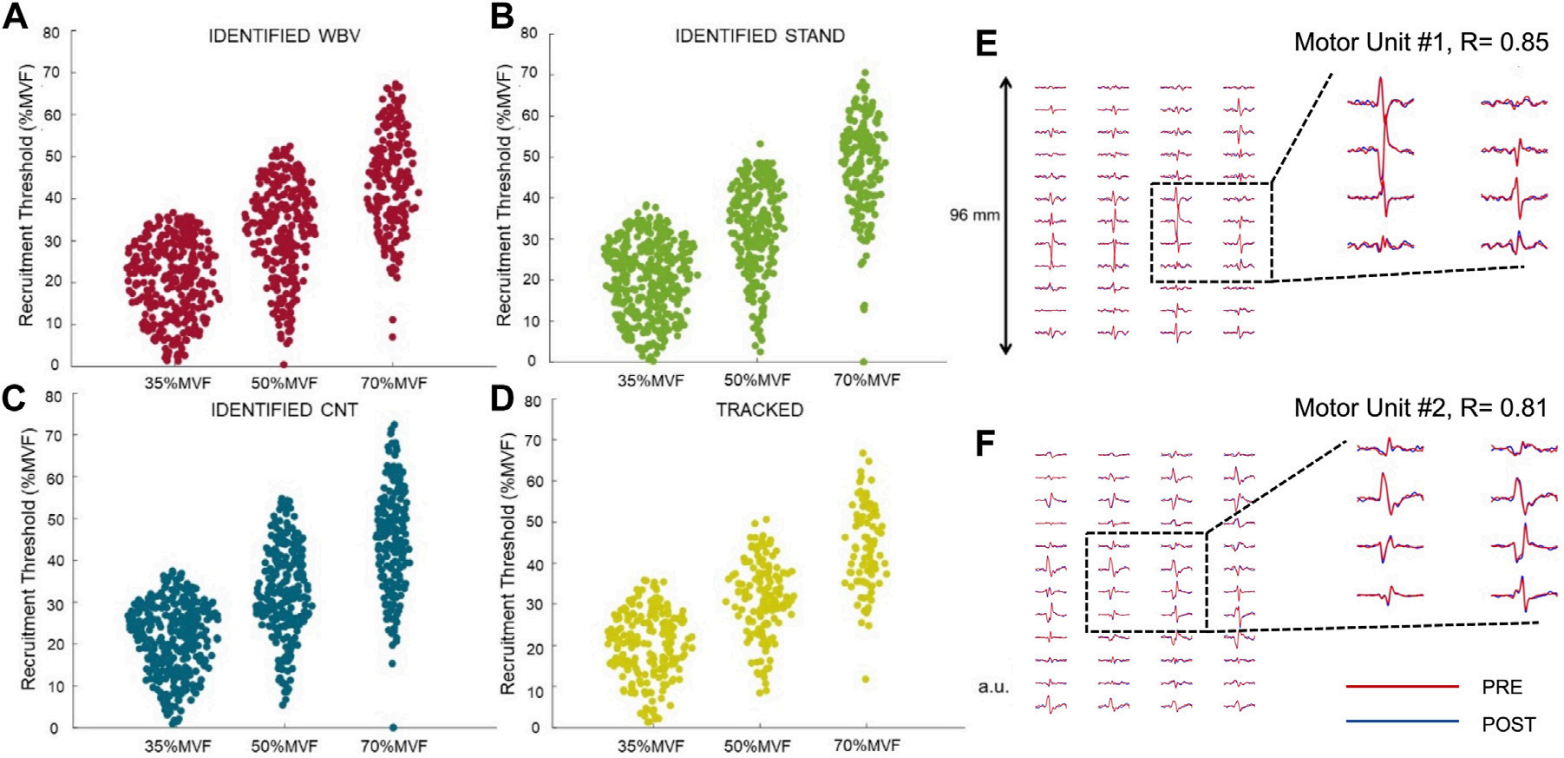
Motor unit tracking analysis. The present figure represents the total identified motor units in WBV **(A)**, STAND **(B)**, and CNT **(C)** classified on the recruitment threshold. Plot **(D)** displays the total number of motor units tracked across conditions classified on the recruitment threshold of the pre-condition. Plot **(E,F)** represent the tracking procedure of two different motor units; the action potential waveforms of the pre-and post-condition, obtained from bipolar high-density EMG signals, were correlated in this procedure. The red and the blue lines are almost overlapped, as a sign of high two-dimensional correlation (r). The columns and rows represent the dimensions of the high-density electrodes.

Only trapezoidal contractions without pre-tension were analyzed. HDsEMG signals were decomposed with the convolutive blind source separation (BSS) method (Holobar and Zazula, 2007). This decomposition procedure can identify MU discharge times over a broad range of forces. An experienced investigator manually analyzed the identified MUs, keeping only those characterized by a high pulse-to-noise ratio (Holobar et al., 2014). MUs with a pulse-to-noise ratio <30 dB or discharge times separated by more than 2 s were excluded from the analysis. The recruitment and derecruitment thresholds (RT and DT, respectively) were identified as the %MVF at which MUs were activated and deactivated, identifying the first and the last spikes, respectively. The average MU discharge rate (DR) was computed from the series of discharge times identified by the decomposition. The average discharge rate for each MU was assessed during the recruitment (REC), plateau (PLA), and derecruitment (DER) phases. The interspike interval variability (ISIvar), which is the variability of consecutive motor unit spikes, was assessed during the plateau phase of the trapezoidal ramps. Data from all target forces are useful for describing differences in the mentioned properties in all motor unit populations (low-, mid-, and high-threshold motor units) (del Vecchio et al., 2019). The motor unit tracking was performed across all conditions (WBV, STAND, CNT) and was used to improve comparison robustness between these. The reliability of the motor unit tracking (Figure 4) is based on the correlation value of the two-dimensional action potential waveforms (del Vecchio et al., 2019; Martinez-Valdes et al., 2017). Only motor units with a high correlation value were taken into analysis (arbitrary R > 0.8).

### 2.7 Statistical analysis

A statistical power analysis was performed *a priori* to determine the sample size (G*power software version 3.1.9.4; α = 0.05, power = 0.80 effect size = 0.4; the total sample size needed was 12). The data normality distribution was assessed using the Kolmogorov- Smirnov test. All the present data showed a normal distribution. Two-way (time: Pre- and Post-intervention; group: CNT, STAND, WBV) ANOVA was used to test differences in the absolute and relative RT, absolute and relative DT, mean overall DR, and at the recruitment, plateau, and derecruitment, and the ISIvar. Since no statistically significant differences were found, no *post hoc* analysis was done. Paired samples T-tests were used to compare the number of identified MUs across conditions and ramp contraction forces. The statistical calculations were performed using SPSS 25.0 (IBM Corp., Armonk, NY, United States). A *p* < 0.05 was considered a statistically significant result. Data are reported as the Mean ± SD in the text.

## 3 Results

### 3.1 MU decomposition and tracking

A total of 2,447 motor units were identified, considering all conditions. Of these, 452 MUs (roughly 18%) were tracked across all the analyzed conditions (WBV, STAND, CNT) at the three target forces (Figure 4). The tracking procedure consisted in identifying the same MU in the Pre- and Post-condition for each target force. The average number of identified MUs did not differ between conditions (*p* = 0.89) but was significantly different between ramp contraction intensities: 35%MVF to 50%MVF (*p* = 0.04), 50%MVF to 70%MVF (*p* = 0.01), 50%MVF to 70%MVF (*p* = 0.005). The reliability of the MU tracking technique is based on the correlation of the two-dimensional action potential waveforms (Martinez-Valdes et al., 2017). The average two-dimensional correlation, for the action-potential waveforms tracked before and after the provided conditions, was 0.86 ± 0.1. Table 1 reports the number of identified motor units with the relative percentage and the absolute number of tracked motor units classified by the %MVF and condition.

**TABLE 1:** Number of identified and tracked motor units.

	Identified motor units			Total	Tracked motor units
	35%MVF	50%MVF	70%MVF		
WBV	333	275	202	810	161 (20%)
STAND	384	247	190	821	149 (18%)
CNT	362	260	194	816	142 (18%)
Total	2447				452

### 3.2 Myoelectrical parameters

**FIGURE 5:**
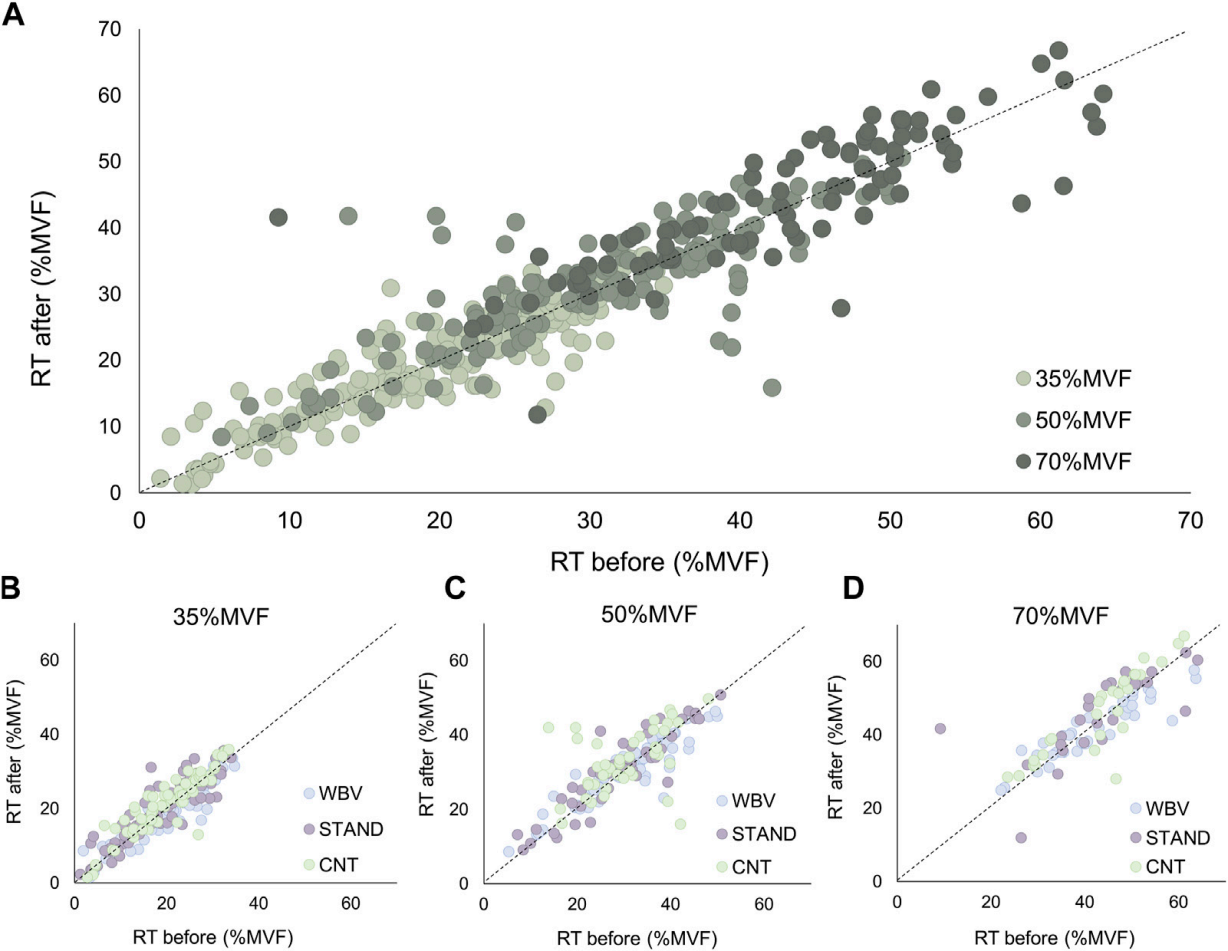
Motor unit recruitment threshold. The scatter plots of the recruitment threshold before (abscissa) and after (ordinate) each condition. It was displayed for the overall results **(A)**, 35%MVF **(B)**, 50%MVF **(C)**, and 70%MVF **(D)**.

Recruitment and discharge properties were analyzed in tracked motor units. Based on the tracked MUs data, in the three submaximal target forces, non-significant effects were observed in the motor unit absolute recruitment threshold for each condition [WBV: 91.81 ± 40.8 to 89.34 ± 39.4; STAND: 81.9 ± 42.8 to 86.49 ± 46.0; CNT: 89.54 ± 38.8 to 95.44 ± 41.7 (N)]. In addition, the normalized recruitment threshold showed no significant effects [WBV: 29.36 ± 12.7 to 28.45 ± 12.2; STAND: 26.19 ± 13.6 to 27.47 ± 13.9; CNT: 28.16 ± 12.1 to 30.02 ± 12.8 (%MVF)]. Scatter plots displayed in Figure 5 represent the differences between the PRE- and the POST-conditions (WBV, STAND, CNT) in tracked motor units.

**FIGURE 6:**
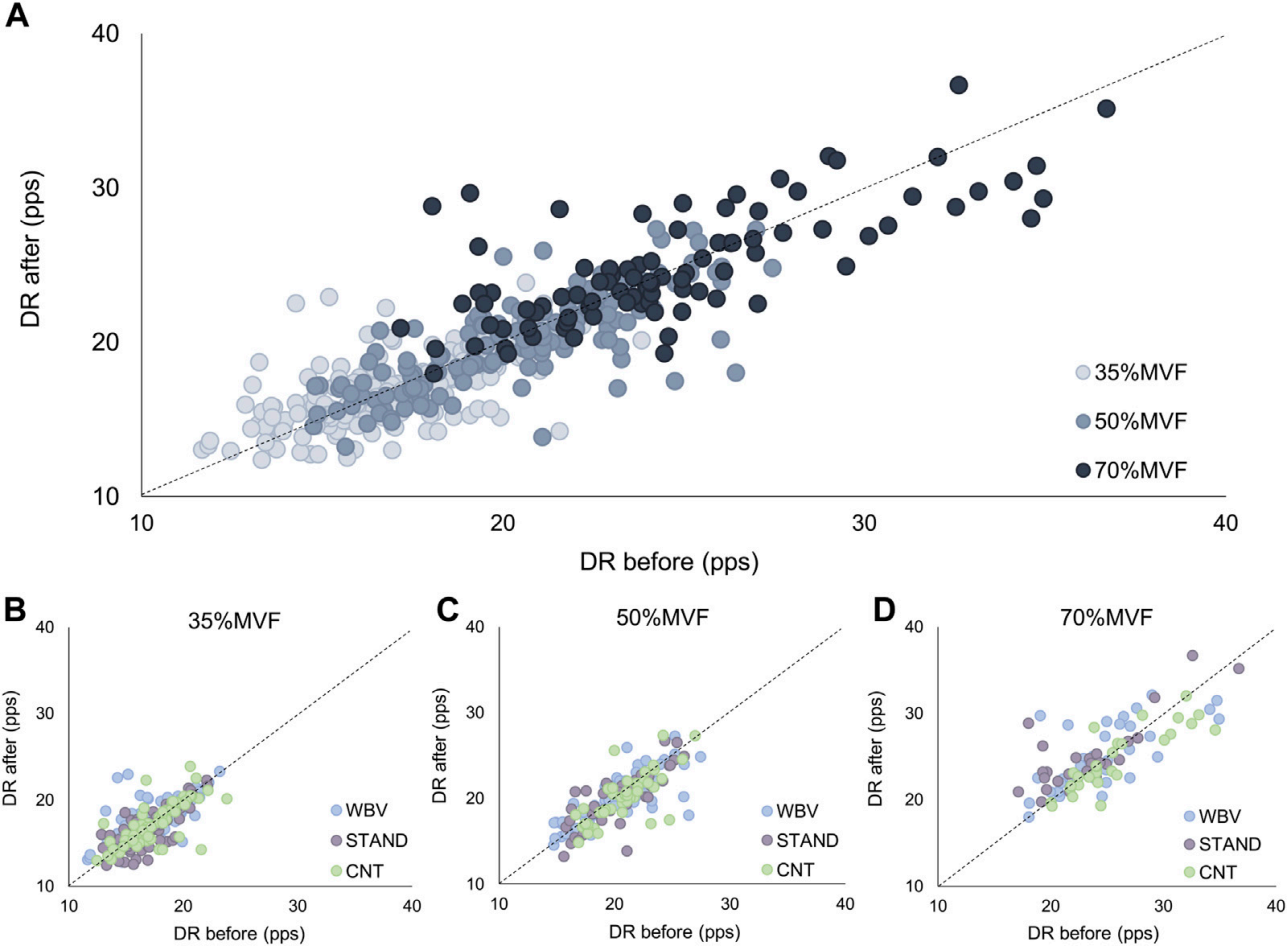
Motor unit discharge rate. The scatter plots of the discharge rate before (abscissa) and after (ordinate) each condition. It was displayed for the overall results **(A)**, 35%MVF **(B)**, 50%MVF **(C)**, and 70%MVF **(D)**.

The distribution along the broken line is representative of non-significant variations in all conditions, confirmed by ANOVA analyses. Concerning the motor unit derecruitment threshold, no significant differences were found in the absolute values [WBV: 101.67 ± 46.4 to 100.27 ± 46.2; STAND: 91.57 ± 49.1 to 93.65 ± 49.2; CNT: 98.8 ± 48.3 to 100.85 ± 46.2 (N)]. Moreover, no significant changes were observed in the normalized derecruitment threshold [WBV: 32.43 ± 14.4 to 31.87 ± 13.9; STAND: 29.02 ± 14.7 to 29.60 ± 14.3; CNT: 31.86 ± 14.3 to 31.48 ± 13.5 (%MVF)]. Additionally, tracked motor units showed non-significant effects in the mean discharge rate across all conditions and target forces [WBV: 19.95 ± 4.5 to 20.24 ± 4.1; STAND: 19.23 ± 3.8 to 19.19 ± 4.2; CNT: 20.10 ± 4.2 to 19.52 ± 3.9 (pps)], as displayed in Figure 6.

**FIGURE 7:**
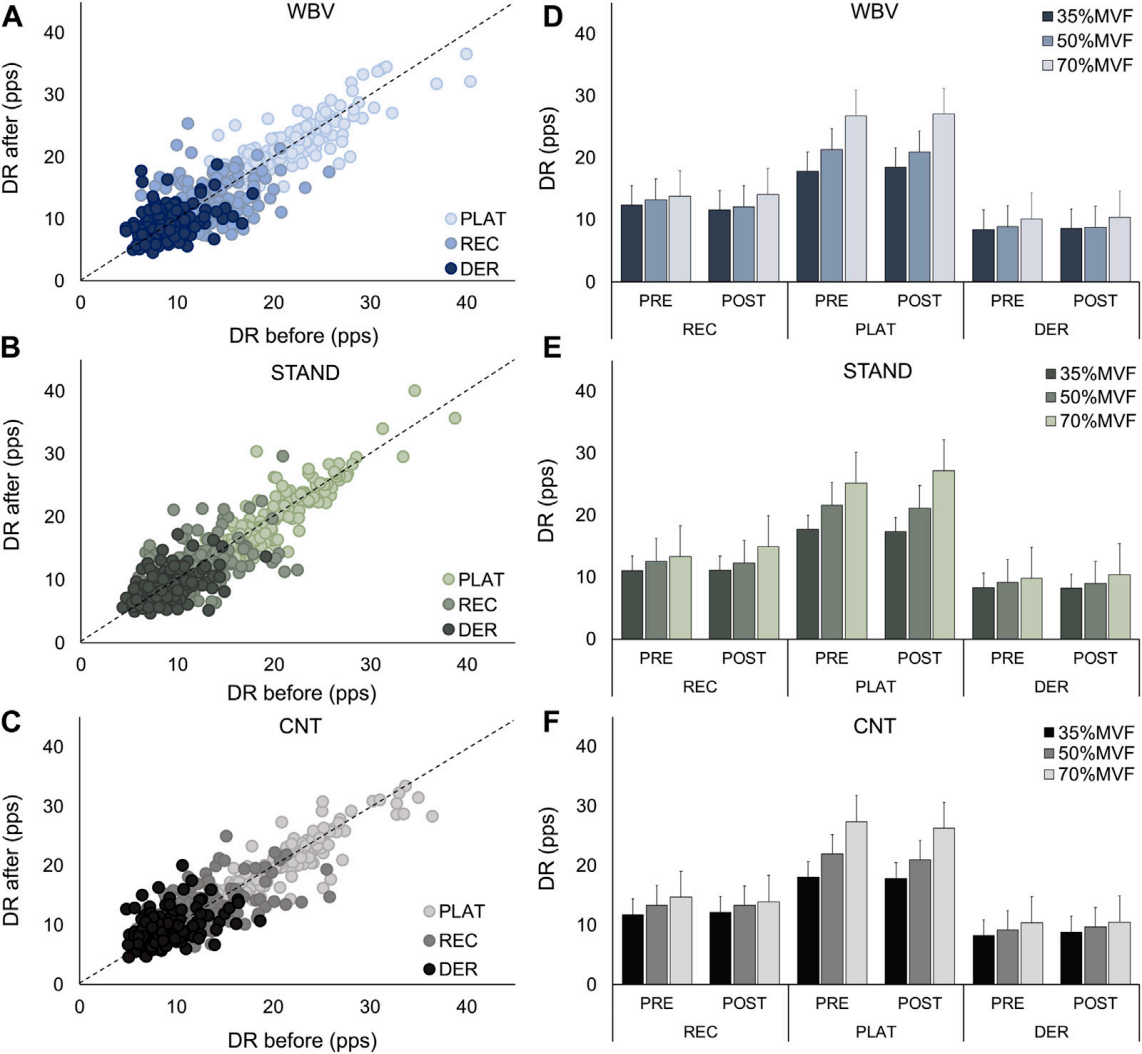
Discharge rate at the recruitment, plateau, and derecruitment. In the left panels, scatter plots of the discharge rate at the recruitment, plateau, and derecruitment for each given target force are displayed, with the discharge rate before (on the abscissa) and the discharge rate after (on the ordinate). Panels **(A–C)** represent WBV, STAND, and CNT conditions respectively. The panels on the right display bar plots of the discharge rate at each target force’s recruitment, plateau, and derecruitment. Panels **(D–F)** represent WBV, STAND, and CNT conditions respectively. Data are reported as mean ± SD.

Furthermore, the mean discharge rate at the recruitment, plateau, and derecruitment was analyzed to determine possible effects on ramp phases. As depicted in Figure 7, the DR showed no significant changes between the Pre- and Post-contractions phases in all conditions (WBV DR [REC: 12.99 ± 3.4 to 12.38 ± 3.5; PLA: 21.14 ± 5.2 to 21.35 ± 4.7; DER: 9.0 ± 2.4 to 9.12 ± 2.5 (pps)]; STAND DR [REC: 11.98 ± 3.4 to 12.20 ± 3.6; PLA: 20.33 ± 4.3 to 20.36 ± 4.8; DER: 8.87 ± 2.4 to 8.82 ± 2.4 (pps)]; CNT DR [REC: 12.83 ± 3.8 to 12.85 ± 3.4; PLA: 21.12 ± 4.7 to 20.51 ± 4.4; DER: 8.95 ± 2.7 to 9.38 ± 2.7 (pps)]). Moreover, the interspike variability (ISIvar) non-significantly changed in all conditions [WBV: 18.85 ± 7.92 to 19.39 ± 7.1; STAND: 17.77 ± 7.7 to 18.94 ± 8.1; CNT: 19.73 ± 9.3 to 20.82 ± 8.4 (%)]. All the previous results showed a *p*-value> 0.05.

## 4 Discussion

The present study underlined the interaction between motor unit properties and acute WBV exposure. In order to understand motor control strategies, recruitment threshold and discharge rate across all motor unit populations were studied. Analyses were performed through HDsEMG, differently from previous studies examining whole-body vibration effects on similar parameters (Cardinale and Bosco, 2003; Kurt and Pekünlü, 2015). However, apart from changes in discharge parameters reported in a single study (Cochrane et al., 2010), there is little evidence about the specific motor unit recruitment and discharge properties following WBV which could explain performance improvements previously reported (Delecluse et al., 2003; Rees et al., 2009). After acute exposure to vibration, greater effects in RT were found in lower threshold MUs, which showed a tendency to decrease. In a previous study, Pollock et al. (2021) have reported increased MURT in lower threshold MUs after acute WBV. However, the authors found a similar result in higher threshold motor units’ RT, confirming a reduced recruitment threshold following acute vibration exposure. Based on our results, acute vibrating stimulus did not elicit significant effects in all motor unit populations investigated. So, it is possible that different vibrating stimuli may be able to elicit changes in the different motor unit populations, however acute WBV provided in the present protocol (30 Hz; 4 mm) was not able to affect the motor unit recruitment threshold analyzed through HDsEMG. The discharge rate, measured in WBV trials motor units, appeared slightly increased at the three target forces, showing a tendency to increase after acute vibration exposure. Nevertheless, the overall discharge rate (DR) and the DR at recruitment appeared slightly decreased, whereas at plateau, and derecruitment showed a non-significant increase after the acute whole-body vibration application across all %MVF. Furthermore, even if no significant differences were found in the discharge rate at the recruitment, plateau, and derecruitment before and after WBV, the distributions across the ramp phases were similar to the results of previous studies investigating the same parameters (del Vecchio et al., 2019; Nuccio et al., 2021a).

**FIGURE 8:**
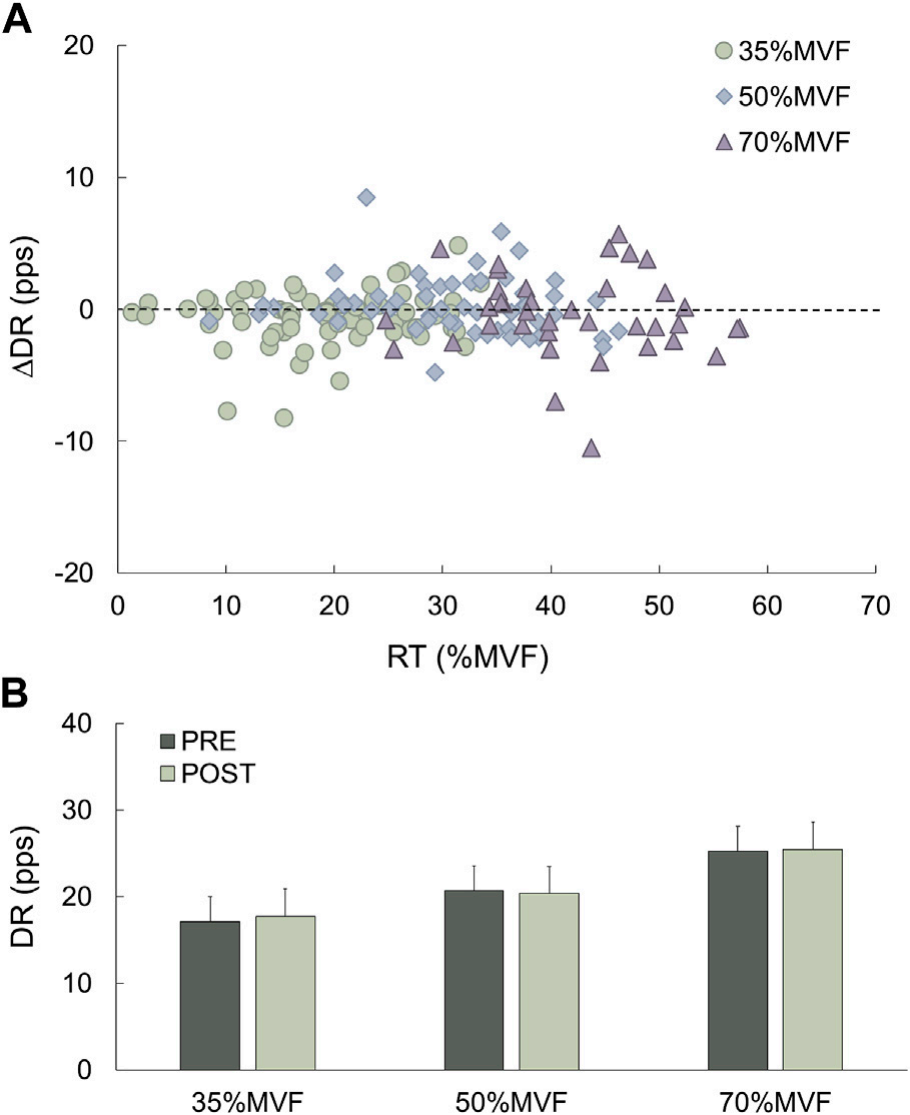
Difference in motor unit discharge rate (Pre- and Post-WBV) relative to normalized recruitment threshold after the exposure. **(A)** The difference in the discharge rate (ordinate) as a function of the recruitment threshold after the intervention in the WBV group. **(B)** Bar plot of the overall discharge rate changes. Data are reported as mean ± SD.

**FIGURE 9:**
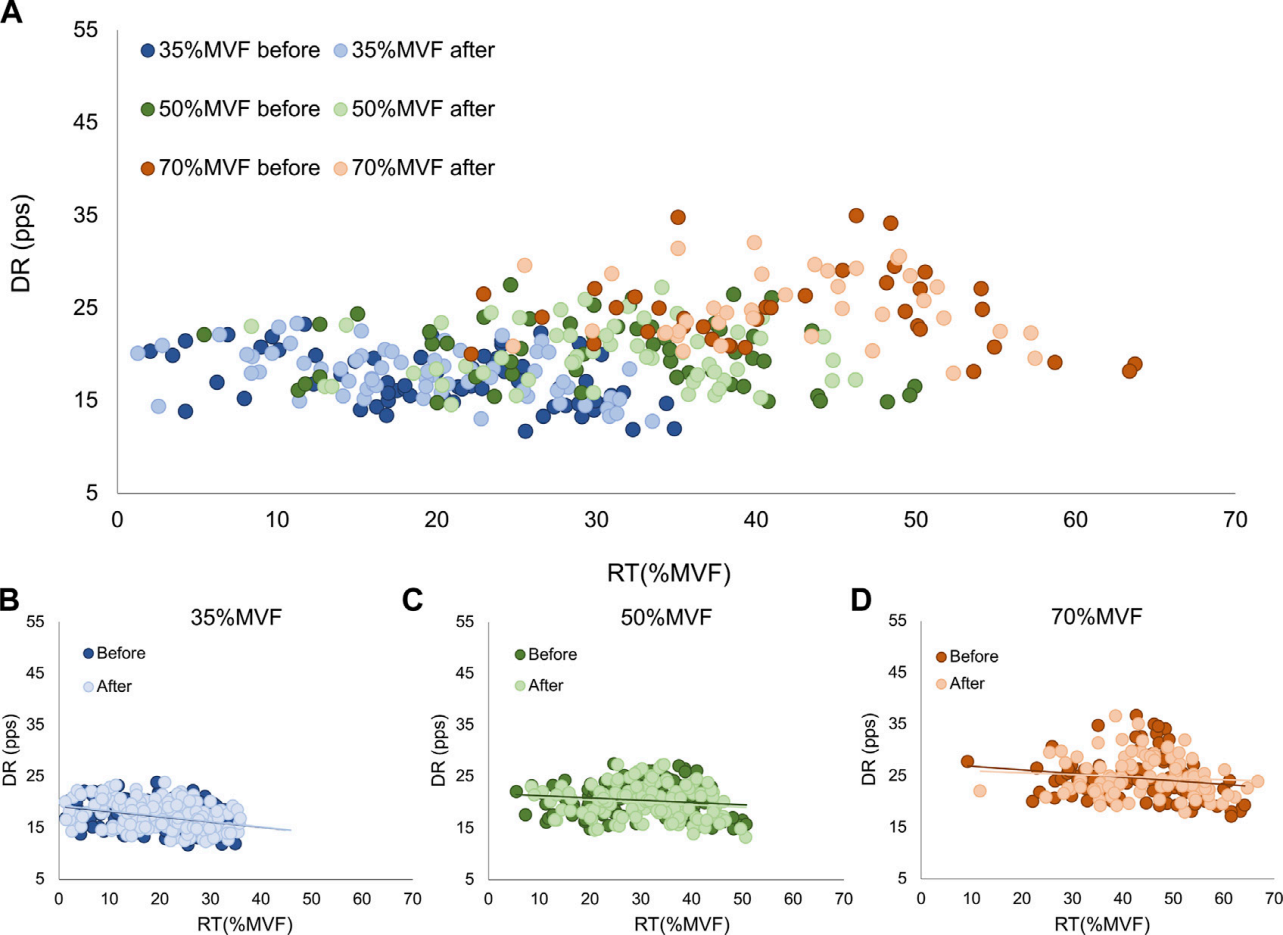
Relationship between discharge rate and recruitment threshold of motor units before and after acute WBV exposure. The discharge rate (ordinate) as a function of the recruitment threshold (abscissa) for representing the relationship between these two parameters as the overall results **(A)**, at 35%MVF **(B)** 50%MVF **(C)**, and 70%MVF **(D)**, in WBV group.

The difference between the DR before and after WBV as a function of the RT after WBV, plotted in Figure 8, shows that the discharge rate in all motor unit populations remained similar. In fact, the distribution along the broken line confirms the statistical analysis, showing no significant differences. Besides, the relationship between the discharge rate and the recruitment threshold was similar after WBV at the three target forces (Figure 9), showing an inverse relationship, as described in previous studies (de Luca and Hostage, 2010), as a further confirmation that neither DR nor the RT were affected by WBV.

According to the present results, acute vibration effects on motor unit properties are not statistically significant, suggesting that neural changes reported in previous studies are not utterly derived from increased neural drive to muscles. This is likely influenced by the isometric conditions used in the present study, which were mandatory for analyzing through HDsEMG. Many authors have found several significant differences in dynamic or plyometric conditions (Cardinale and Bosco, 2003; Delecluse et al., 2003; Cochrane et al., 2010; Kurt and Pekünlü, 2015) after repeated WBV exposure in a single session, whereas in the present study, data were acquired necessarily in isometric conditions due to the use of HDsEMG (Holobar et al., 2009). Changes in contraction conditions may have affected the result, even if analyzing motor unit properties through HDsEMG is useful to directly comprehend motor unit parameter variations, in other words, motor neuron properties. No studies have reported changes in muscle properties after acute whole-body vibration, so, it is unlikely that changing contraction conditions affected motor neuron properties in response to acute WBV, in non-fatiguing tasks. Thus, the present results showed no significant effects in motor neuron properties after acute WBV. Furthermore, no studies have investigated WBV effects on motor unit properties through HDsEMG, reporting only classical EMG results, which may have affected the outcome of the present analysis. Tracking the same motor units across a given period and identifying a single motor unit in a row may be necessary for assessing specific neuromuscular parameters. Previous results described the overall neural changes providing purely an overall insight into the neuromuscular changes. Nevertheless, HDsEMG has been widely demonstrated to be an advanced technique, being able to solve part of the problem, with its limit of recording during isometric contractions, which may have brought different results than previous analyses conducted on dynamic or plyometric conditions. Other possible explanations may be related to cortical activity, which can be affected by the afferent signals that have been speculated to be affected by WBV (Cochrane et al., 2010).

The main limitation in the present study is represented by the necessity for isometric conditions recordings to correctly use the HDsEMG, to analyze single MUs across a given period (Holobar et al., 2014; Farina et al., 2016). Moreover, the present study aimed to describe the effects of WBV on motor unit properties, which limited the exploration performance parameters, such as repeated MVCs, due to potential effects on the acquiring signal. In is likely that spinal and supraspinal circuitries are affected by vibrating stimuli. Further studies investigating acute WBV on neuromuscular parameters, associating both peripheral and central parameters would bring novelty and knowledge to science.

## 5 Conclusion

In conclusion, the present results provide evidence that acute exposure to WBV (30 Hz; 4 mm; 1 min of exposure) does not elicit variations concerning motor unit recruitment, and discharge properties, in trapezoidal isometric contractions. Further studies involving both brain and spinal output measurements may provide novel findings on neural changes after acute or chronic exposure to WBV, in both isometrics and dynamics.

## Data Availability

The raw data supporting the conclusions of this article will be made available by the authors, without undue reservation.
